# SARS-CoV-2 S Protein Subunit 1 Elicits Ca^2+^ Influx – Dependent Ca^2+^ Signals in Pancreatic Stellate Cells and Macrophages *In Situ*

**DOI:** 10.1093/function/zqac002

**Published:** 2022-01-31

**Authors:** Julia V Gerasimenko, Ole H Petersen, Oleg V Gerasimenko

**Affiliations:** School of Biosciences, Sir Martin Evans Building, Cardiff University, Wales CF10 3AX, UK; School of Biosciences, Sir Martin Evans Building, Cardiff University, Wales CF10 3AX, UK; School of Biosciences, Sir Martin Evans Building, Cardiff University, Wales CF10 3AX, UK

**Keywords:** SARS-COV-2, S1 spike protein, calcium signalling, pancreatic macrophages, pancreatic stellate cells, interleukin-18, IL-18BP, ORAI1 CRAC channels, CM4620

## Abstract

The S protein subunit 1 (S1) of SARS-CoV-2 is known to be responsible for the binding of the virus to host cell receptors, but the initial intracellular signalling steps following receptor activation of cells in the exocrine pancreas are unknown. Using an intact live mouse pancreatic lobule preparation, we observed that S1 elicited Ca^2+^ signals in stellate cells and macrophages, but not in the dominant acinar cells. The Ca^2+^ signals occurred mostly in the form of repetitive Ca^2+^ spikes. The probability of observing Ca^2+^ signals depended on the S1 concentration. The threshold was close to 70 nM, whereas at 600 nM, all cells responded. The SARS-Cov-2 nucleocapsid protein did not elicit any Ca^2+^ signals in any of the three cell types tested. The S1-induced Ca^2+^ signals in stellate cells started much faster (122 ± 37s) than those in macrophages (468 ± 68s). Furthermore, the interleukin-18 binding protein (IL-18BP) abolished the responses in macrophages without affecting the Ca^2+^ signals in stellate cells. The S1-elicited Ca^2+^ signals were completely dependent on the presence of external Ca^2+^ and were abolished by a selective inhibitor (CM4620) of Orai1 Ca^2+^ Release Activated Ca^2+^ channels. SARS-CoV-2 may contribute to acute pancreatitis, an often fatal inflammatory human disease. The S1-elicited Ca^2+^ signals we have observed in the pancreatic stellate cells and endogenous macrophages may play an important part in the development of the inflammatory process.

## Graphical Abstract

**Graphical Abstract:**
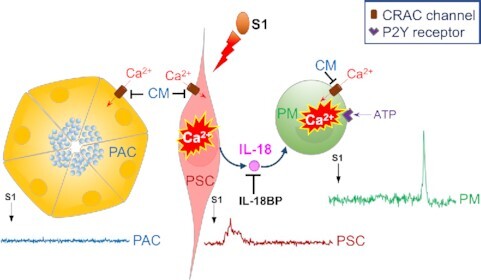
SARS-CoV-2 S Protein Subunit 1 Elicits Ca^2+^ Influx – Dependent Ca^2+^ Signals in Pancreatic Stellate Cells and Macrophages In Situ.

## Introduction

SARS-CoV-2 enters cells via receptor-mediated endocytosis^1^, using binding of its S protein subunit to ACE2 receptors thereby allowing endocytic uptake of the virus^2^. SARS-CoV-2 is known to enter a number of cell types in the human body including the respiratory tract^2^, the central nervous system^3^ and the gastro-intestinal tract, including the exocrine pancreas^4^, with several reported cases of acute pancreatitis (AP)^4^. The ACE2 receptor is present in the pancreas^4^, but the mechanism by which SARS-CoV-2 can induce AP as well as the type of cells targeted by the virus are currently unknown.

Pancreatic acinar cells dominate the exocrine pancreas and are largely responsible for the initiation of AP^5^. However, other cell types in this tissue also play important roles, in both physiological and pathophysiological processes, including AP^5–11^. Our novel method of using isolated lobules of the exocrine pancreas^7,9,10^, allowing preservation of the normal microscopic structure of the pancreatic environment, enables simultaneous recordings of intracellular signals i.e., cytosolic Ca^2+^^6–11^ and nitric oxide^11^ in several different cell types, including stellate cells^9^, intrinsic nerves^10^ and pancreatic macrophages^7^.

Whereas physiological and pathological Ca^2+^ signals in pancreatic acinar cells are well understood^5^, their roles in the stellate cells^9^ and the macrophages^7^ are less clear. There is, however, evidence that Ca^2+^ and NO signals in stellate cells amplify the pathological effects in AP^8–11^. In our recent work^7^ we have studied immune cell Ca^2+^ signalling in response to stimulation with ATP and identified the responding cells as macrophages by immunohistochemistry with external markers specific for this cell type^12–14^. Macrophages are involved in the development of both AP and pancreatic cancer^15^, leading to inflammation and exacerbation of the patients’ conditions^16^, but the evidence for this has largely been limited to work on cell cultures or fixed tissue samples of the pancreas. Taking advantage of the pancreatic lobule preparation, we now report for the first time Ca^2+^ signals induced by the SARS-CoV-2 S1 protein in pancreatic cells.

## Methods

### Animals

Ethical Approval: All animal studies were ethically reviewed and conducted according to the UK Animals (Scientific Procedures) Act, 1986. All experimental protocols were performed under a Project Licence granted by the UK Home Office and approved by the Animal Care and Ethics Committees at Cardiff School of Biosciences, Cardiff University. Animals were maintained in plastic cages supplied with fresh corn cob bedding, tap water, and commercial pelleted diet.

### Lobule Preparation

Pancreatic lobules were freshly isolated from the pancreas of 5- to 7-week-old male C57BL6/J mice as previously described^7^, ^10^. The pancreas was rapidly removed, injected with standard Na^+^-Hepes-based solution containing collagenase and incubated for 5–6 min at 37°C. The standard solution was composed of (in mM): NaCl, 140; KCl, 4.8; Hepes, 10; MgCl_2_, 1; glucose, 10; CaCl_2_, 1 (unless stated otherwise), pH 7.3 (NaOH). All experiments were carried out with pancreatic lobules attached to the coverslip of a perfusion chamber at room temperature (22°C).

### **Ca^2^^+^** Measurements

Pancreatic lobules were loaded with Fluo-4 acetoxymethyl (AM) ester at room temperature as described previously^7^. The lobules were transferred into a flow chamber and perfused with the standard solution alone or containing different chemicals as described in the experimental protocols of the result section. Cells were visualized using a Leica SP5 MPII two-photon confocal microscope, with an x63 1.2NA objective lens. The Fluo-4 excitation wavelength was 488 nm and emission was collected at 500–560 nm with resolution of 256 × 256 pixels and speed of 0.7 frames/s. Images were analysed using Leica Confocal Software (Leica, Mannheim, Germany). Fluorescence signals were plotted as normalized F/F0. ANOVA or Student's t-test were performed for statistical analysis.

### Immunostaining in *ExVivo* Pancreatic Lobules

Immunostaining of live pancreatic lobules was performed as previously described^7^. Mouse F4/80 Alexa Fluor 647-conjugated monoclonal rat antibodies were used to label specific surface proteins of immune cells, usually at the end of Ca^2+^ measurement experiments, unless otherwise stated. After blocking with 1% BSA and 10% goat serum containing PBS, the isolated pancreatic lobules were incubated for 1 h at room temperature with the selected antibody. Antibody staining was visualized by exciting Alexa Fluor 647 with 633 nm laser at 10% power and emitted light was collected at 640–700 nm. Conjugated antibody fluorescence was also overlaid with Fluo-4 staining as described in the Ca^2+^ measurements section. Lobules were attached to the glass coverslips covered with poly-L-lysin.

### Materials

SARS-CoV-2 S1 protein and Nuclear Capsid protein were purchased from GenScript or Abcam; S1 RBD (Receptor Binding Domain) was obtained from Abcam. IL-18BP was from R&D. Bradykinin (BK) was purchased from Tocris Biosciences (UK). Fluo-4 AM and Hoechst 33342 were purchased from ThermoFisher Scientific (UK). Anti-mouse F4/80 monoclonal rat antibody (CI-A3-1) [Alexa Fluor 647] were obtained from Novus Biologicals. CM4620 was provided by CalciMedica Inc., US. Other chemicals were purchased from Sigma or Calbiochem (Merck, UK).

## Results

**Figure 1.**
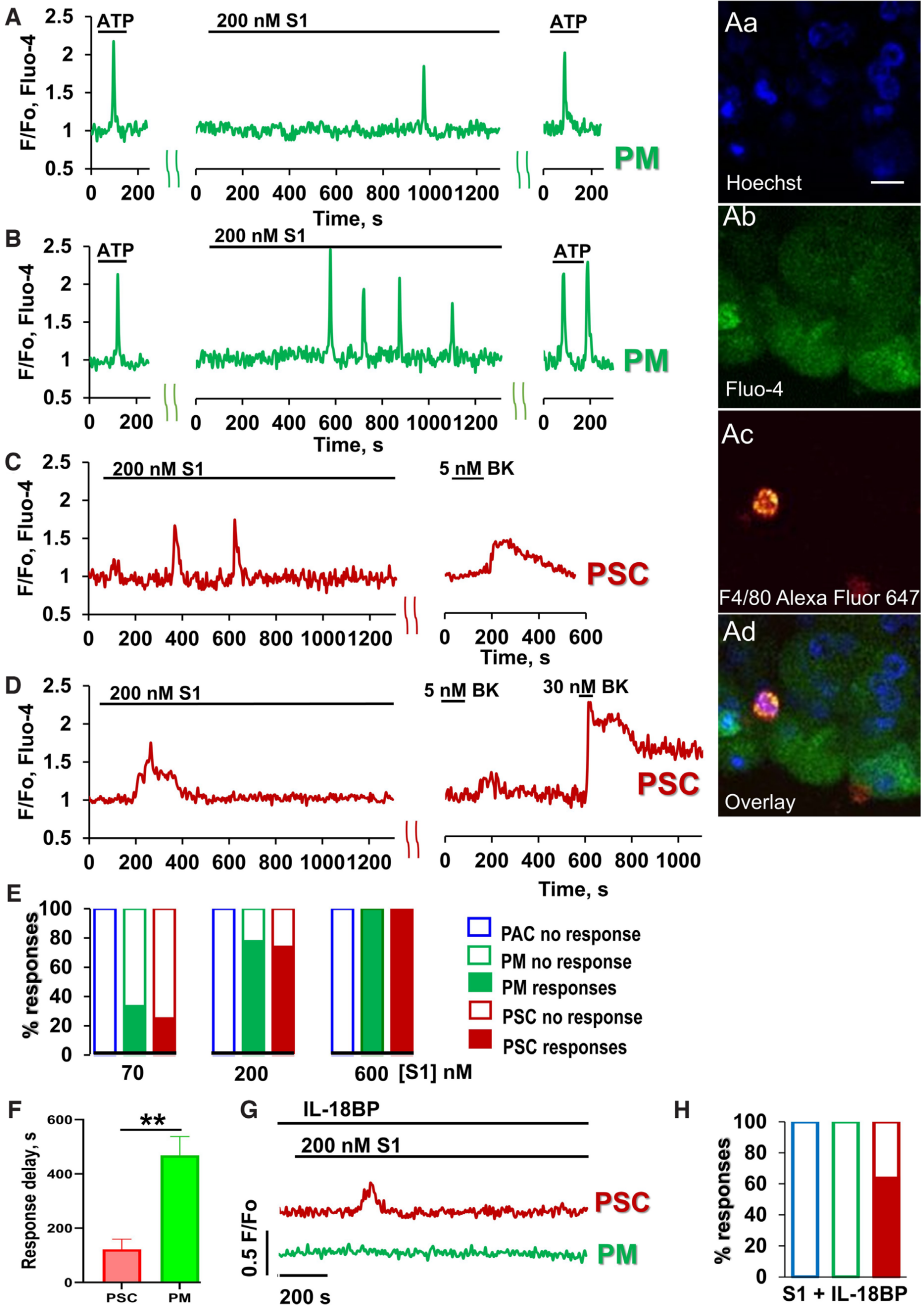
Pancreatic immune and stellate cells exhibit Ca^2+^ signals in response to the addition of SARS-CoV2 S1 protein. A-B. Representative traces demonstrate single and multiple Ca^2+^ signals from pancreatic immune cells in response to S1 addition (Pancreatic macrophages (PM), green traces). Lobules were stimulated with 2.5 μM ATP, followed by application of 200 nM S1 (for 20 min). At the end of each experiment 2.5 μM ATP was consecutively added (n = 50). At the end of Ca^2+^ measurements, lobules were stained under the microscope with fluorescently labelled antibodies for macrophages (F4/80) and nuclear staining with Hoechst 33342. Images of nuclear staining (Hoechst 33342, Fig 1Aa), calcium dye (Fluo-4, Fig 1Ab) and antibody staining (AlexaFluo 647, F4/80, Fig 1Ac) for the same area as for the trace in Fig 1A (images in Fig 1Aa-c with overlay presented in Fig 1Ad; scale bar 10µm). C-D. Representative traces of single (C) and multiple (D) Ca^2+^ responses from pancreatic stellate cells (PSC, red traces) in pancreatic lobules. Lobules were stimulated with 200 nM S1 for 20 min. At the end of each experiment 5 nM and/or 30 nM BK were sequentially added (n = 23). (E) Concentration-dependence of PSCs and PMs responding to S1 stimulation by Ca^2+^ signals (red and green data bars, respectively). No responses were observed from acinar cells (PAC, blue data bars). The number of responses was calculated including single and multiple Ca^2+^ spikes to different concentrations of S1 (70 nM, 200 nM, 600 nM). (F) Time between application of S1 (200nM) and beginning of elevation of the cytosolic Ca^2+^ concentration in PSCs (122 ± 37s) and PMs (468 ± 68s). PSCs responded significantly faster, *P*< 0.006. (G) Representative responses to S1 in the presence of IL-18 binding protein (IL-18BP, ) in PMs and PSCs. (H) No Ca^2+^ signals to S1 were observed in the presence of IL-18BP (600nM) in PMs and PACs (n = 7 and n = 33, respectively) while 63.5% of PSCs did respond (n = 11), similarly to results shown in Fig 1E.

**Figure 2.**
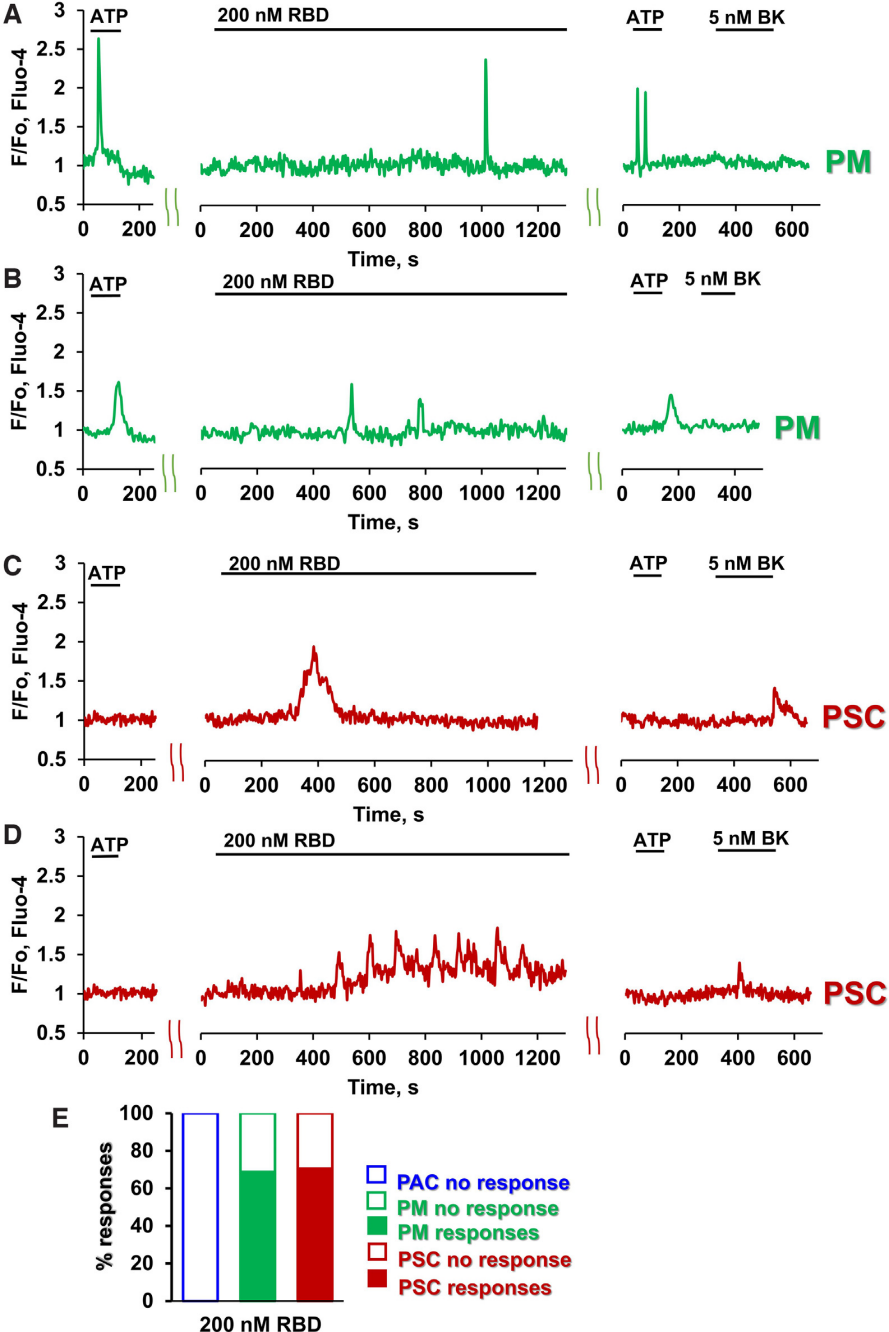
SARS-CoV2 RBD protein elicits Ca^2+^ signals in macrophages and stellate cells. A-B. Representative traces demonstrate single and multiple Ca^2+^ signals from PMs in response to RBD addition (PM, green traces). Lobules were stimulated with 2.5 μM ATP followed by application of 200 nM RBD (for 20 min). At the end of each experiment 2.5 μM ATP and 5 nM BK were consecutively added (n = 16). C-D. Representative traces of single (C) and multiple (D) Ca^2+^ spikes from PSCs (red traces). Lobules were stimulated with 200 nM RBD for 20 min. At the end of each experiment 2.5 μM ATP and 5 nM BK were sequentially added (n = 17). (E) The percentage of cells responding to 200 nM RBD with single or multiple Ca^2+^ signals. PACs, blue; PMs, green and PSC, red.

As previously reported^7,10^, pancreatic macrophages are typically sensitive to ATP or ADP and display Ca^2+^ signals when stimulated by these agents, whereas pancreatic stellate cells are typically insensitive to ATP, but are always responsive to bradykinin (BK), displaying Ca^2+^ signals when stimulated by the nona-peptide. Pancreatic acinar cells are always sensitive to acetylcholine and cholecystokinin, but never respond to stimulation with BK. They can occasionally display Ca^2+^ signals in response to stimulation with ATP. Nevertheless, ATP-sensitive cells are most likely to be macrophages and this can be verified after the recording of Ca^2+^ signals by staining with fluorescently labelled antibodies for macrophages (F4/80) (Figure 1A–D). In the macrophages, untagged S1 protein (200 nM) elicited either a single Ca^2+^ spike (Figure 1A, 38% of cells tested, n = 17) or a train of such spikes (Figure 1B, 40% of cells tested, n = 18). Similar results were obtained from the BK-sensitive stellate cells (Figures 1C, D, n = 23). A lower concentration of S1 (70nM) evoked Ca^2+^ signals in a smaller proportion of cells (33% of macrophages and 25% of stellate cells, Figure 1E), whereas a higher concentration of S1 (600nM) induced Ca^2+^ signals in every stellate cell and macrophage tested (Figure 1E). The latency after S1 application, before the first Ca^2+^ signal appeared, was much longer in the case of the macrophages than for the stellate cells (Figure 1F, *P*< 0.006). The S1-elicited Ca^2+^ signals in macrophages were abolished by the IL-18 binding protein IL-18BP (Fig 1G, H), whereas the responses to S1 in the stellate cells were unchanged (63.5%, n = 11). S1 never elicited any Ca^2+^ signals in the acinar cells (n > 100). We have also tested the effect of the S1 receptor binding domain (RBD). As seen in Figure 2A–E, RBD at a concentration of 200 nM, elicited Ca^2+^ signals in 69% of the macrophages (n = 16) and 71% of the stellate cells tested (n = 17).

We did not observe any spontaneous (in the absence of S1 or RBD) Ca^2+^ signals in any of the stellate cells or macrophages tested (Supplementary Figure A and B; n = 4 and n = 9, respectively). In another series of control experiments, the potential effects of SARS-CoV-2 nuclear capsid protein (200 nM) was tested, but this protein never induced any Ca^2+^ signals in any of the cell types (Supplementary Figure C and D; n = 8 and n = 5, respectively).

**Figure 3.**
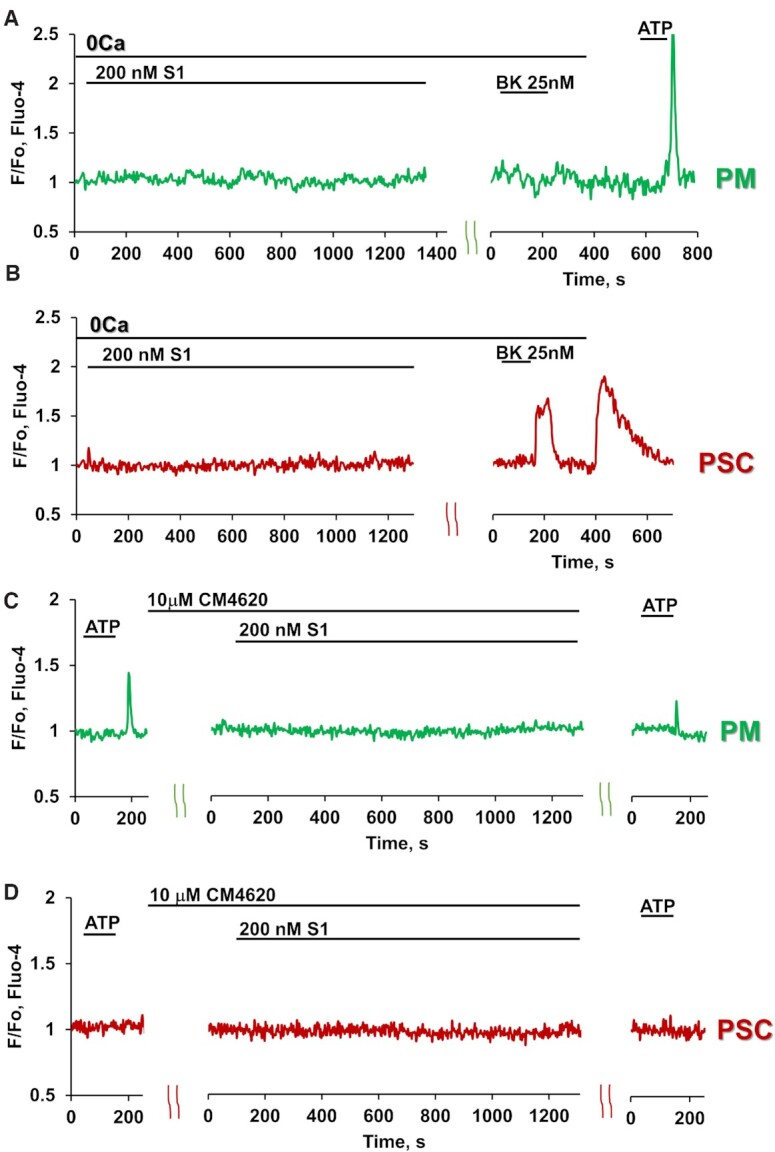
Removal of external Ca^2+^ or application of the Orai1 inhibitor CM4620 block S1-induced Ca^2+^ signals in pancreatic immune and stellate cells. A-B. Representative traces showing that there were no Ca^2+^ signals in response to 200 nM S1 in PMs (n = 11, green trace) and PSCs (n = 9, red trace) in the absence of external Ca^2+^. 25 nM BK and 2.5 μM ATP were added at the end of each experiment. C-D. Representative traces demonstrating the absence of Ca^2+^ signals in response to 200 nM S1 in PMs (n = 11, green trace) and PSCs (red trace; only 2 out of the 8 tested cells responded with Ca^2+^ signals of reduced amplitude and duration), when treated with the Orai1 CRAC channel inhibitor CM4620 (10 μM). 2.5 μM ATP was added at the end of each experiment.

We investigated whether the S1-elicited Ca^2+^ signals were dependent on the presence of Ca^2+^ in the external solution. In the absence of external Ca^2+^, as seen in Figures 3A and B, S1 did not elicit any change in the cytosolic Ca^2+^ concentration in macrophages that were responsive to ATP (n = 11) or in stellate cells (n = 9) that displayed Ca^2+^ signals in response to stimulation with BK. In both these cell types, it has previously been shown that an inhibitor of the opening of Orai1 Ca^2+^ Release Activated Ca^2+^ (CRAC) channels markedly reduces the duration of Ca^2+^ signals evoked by ATP or BK^7^, ^9^. As shown in Figure 3C and D, the Orai1 inhibitor CM4620 abolished S1-elicited Ca^2+^ signal generation in the macrophages (n = 11) and markedly inhibited or abolished Ca^2+^ signals in 75% of the stellate cells tested (n = 8).

## Discussion

There is general agreement that ACE2, the SARS-CoV-2 receptor, is expressed in the exocrine pancreas, but little is known about its distribution between the different cell types^17–19^. Specifically, there is currently no information on the presence or absence of ACE2 in the pancreatic stellate cells or macrophages. Our finding that S1 and its RBD elicits Ca^2+^ signals in both stellate cells and macrophages provides the first evidence for functional receptors, but not necessarily on both cell types. The much longer delay in the response to S1 in the macrophages (Figure 1F), as compared to the stellate cells, might indicate an indirect action of S1 on the macrophages. This conclusion is strengthened by the experiments (Figure 1G) showing that the IL-18 binding protein IL-18BP abolished S1-elicited Ca^2+^ signal generation in the macrophages but not in the stellate cells. The simplest explanation would be that S1-elicited Ca^2+^ signals in the stellate cells stimulate IL-18 secretion, which then in turn elicits Ca^2+^ signals in the macrophages. Stellate cells are capable of IL-18 production and, in the case of chronic pancreatitis, it has been shown that levels of IL-18 are increased following activation by lipopolysaccharide (LPS) or TGF-ß^20^. Although plausible, and compatible with our data, the hypothesis that the S1-elicited Ca^2+^ signals in the macrophages are mediated by IL-18 from the stellate cells needs to be tested further in future experiments. The absence of Ca^2+^ signals in the acinar cells in response to S1 does not rule out the existence of receptors on these cells, as receptor activation could elicit effects other than Ca^2+^ signals but, so far, there are no data providing evidence for functional receptors on the acinar cells.

At this stage there is no information about the intracellular steps leading to the generation of the Ca^2+^ signals, but the fact that these signals, in contrast to those evoked in the same cells by ATP or BK, are completely dependent on the presence of external Ca^2+^ and can be inhibited by the Orai1 CRAC channel inhibitor CM4620, suggests a mechanism of generation that is somewhat different from that of the physiological agonists.

Based on our previous studies of Ca^2+^ and NO signalling in pancreatic stellate cells^9^, ^10^ and Ca^2+^ signalling in pancreatic macrophages^7^, we have proposed that AP, although initiated in the acinar cells, progresses to a severe and often fatal disease state through necrotic amplification loops, sustained by excessive Ca^2+^ entry through CRAC channels in stellate cells, macrophages and acinar cells. We have specifically provided evidence for a number of interaction pathways between acinar cells and stellate cells and between acinar cells and macrophages^5^. The new data presented here suggest a further pathway, not hitherto recognized, between stellate cells and macrophages in which IL-18–secreted by stellate cells in response to activation of SARS-CoV-2 receptors—acts on macrophages to generate Ca^2+^ signals in these cells. IL-18 levels are elevated in AP, and it has previously been suggested that this agent may play a role in acute and chronic pancreatitis together with several other interleukins^21–23^.

The S1 and RBD-elicited Ca^2+^ signals in macrophages and stellate cells, demonstrated in this study, would be likely to exacerbate an otherwise mild case of AP initiated, for example, by the primary action of bile acids or a combination of ethanol and fatty acids on the acinar cells, and could help to explain the known cases of severe AP associated with SARS-CoV-2 infection^4^, ^24^. Our finding that the S1- and RBD-elicited Ca^2+^ signals can be inhibited by an Orai1 CRAC channel blocker, is a further argument in favour of this pharmacological treatment against AP^5^, ^25^.

## Data Availability

The data underlying this article will be shared on reasonable request to the corresponding author.
